# Leach-proof magnetic thrombolytic nanoparticles and coatings of enhanced activity

**DOI:** 10.1038/srep28119

**Published:** 2016-06-20

**Authors:** Andrey S. Drozdov, Vasiliy V. Vinogradov, Ivan P. Dudanov, Vladimir V. Vinogradov

**Affiliations:** 1 ITMO University, Laboratory of Solution Chemistry of Advanced Materials and Technologies, Lomonosova St. 9, 191002, St. Petersburg, Russian Federation; 2 Mariinskiy Hospital, Regional Cardiovascular Center, Liteyny Ave. 56, 191054, St. Petersburg, Russian Federation

## Abstract

Despite the fact that magnetic thrombolytic composites is an emerging area, all known so far systems are based on the similar mechanism of action: thrombolytic enzyme releases from the magnetic carrier leaving non-active matrix, thus making the whole system active only for a limited period of time. Such systems often have very complex structure organization and composition, consisting of materials not approved for parenteral injection, making them poor candidates for real clinical trials and implementation. Here we report, for the first time, the production of thrombolytic magnetic composite material with non-releasing behavior and prolonged action. Obtained composite shows good thrombolytic activity, consists of fully biocompatible materials and could be applied as infinitely active thrombolytic coatings or magnetically-targetable thrombolytic agents.

Atherothrombosis is the principal cause of death worldwide. Heart attacks and strokes are usually acute diseases (i.e., the most severe complications of atherosclerosis) and occur mainly as a result of a blockage that prevents blood flow to the heart or brain^1^. One of the least traumatic and relatively efficient methods to combat thrombosis is a thrombolytic therapy based on enzymatic formulations^2^. However, rapid degradation in the body (usually 5–15 minutes) and drawback effects, such as blood pressure reduction (in about 40% of cases) and development of hemorrhagic complications (in about 10% of cases), including lethal outcome^3^, limits their application. All of these aspects formed the basis for the development of new thrombolytic systems with targeted and prolonged effect.

The greatest popularity among such transport systems was gained by magnetic nanoparticles based on biocompatible magnetite and maghemite^4^,^5^,^6^. Such compositions can be localized at a given point by means of a strong external magnetic field. However, all of these approaches stem from the fact that thrombolytic enzyme is released over time, eliminating the possibility of further magnetic manipulation. In particular, the approaches of co-entrapping magnetic nanoparticles and thrombolytic enzymes in biodegradable matrices of polyethylene glycol^7^, polyacrylic acid^8^ or albumin^9^ were previously studied. Alternatively, core-shell type thrombolytic nanostructures implying prolonged release of an enzyme providing therapeutic effect^10^,^11^,^12^,^13^ were developed as well. Other methods consider covalently cross-linking enzymes with a magnetic carrier surface^14^,^15^,^16^, but in this case a thrombolytic enzyme is rapidly inactivated, since it remains vulnerable to the body’s immune system. Additionally, such cross-linking leads to a change in the structure of the native enzyme and results in a decrease in its activity.

In our previous studies, we started to develop a new concept for the production of thrombolytic sol-gel materials^17^, by the direct entrapment of thrombolytic enzyme into boehmite sol-gel matrix. By using this approach it is possible to obtain nanocomposites capable of functioning for a long time without loss of activity, since a therapeutic enzyme is unable to release from a matrix, being protected from adverse external factors, but it is able to perform its catalytic functions by successfully interacting with the molecules of plasminogen, converting it into plasmin which is relatively active with respect to fibrin clots. Such nanocomposite organisation proved to be very promising for creation of stationary thrombolytic cover, such as thrombolytic coatings for vascular graft^18^. For further development of this concept, we put a simple question: Is it possible to create magnetically controlled thrombolytic nanoparticles with prolonged effect organized by the same principle?

In this article, we present a procedure for the preparation of the thrombolytic magnetic nanoparticles produced by entrapment methodology and provide information on their properties for the first time. Nanocomposites were obtained (for details, see experimental part) via co-condensation of urokinase-type plasminogen activator (uPA), which is commonly used in clinical practice, and magnetite sol prepared by the recently published method implying the production of high purity magnetite sol without using stabilizers and additives^19^. As a result of irreversible sol-gel transition, a xerogel nanoporous matrix forms, whose nanopores entrap an enzyme, and such a matrix can either be used as an enzymatically active coating or crushed to yield bioactive nanocomposite particles^20^. The matrix itself is made up of regular-shaped truncated octahedra with an average diameter of 10 nm (Fig. 1a), possessing well-developed porous nanoarchitecture and a specific surface area of 120 m^2^/g (see ESI Fig. 1S for details of N_2_ physisorption data). Due to controlled co-condensation of uPA and a magnetite matrix, organized nanostructure forms, in which the enzyme molecules may be located in the depth of the matrix or in the surface and subsurface layer. According to our previous observations^18^, subsurface location allows the enzyme to interact with the macromolecular substrate, in our case plasminogen, preventing its release from the matrix at the same time.

In the design of the thrombolytic nanocomposite urokinase-type plasminogen activator was selected because of its structure and mechanism of action. This class of plasminogen activators consists of three distinct domains and can convert plasminogen to plasmin directly by cleaving the Arg561-Val562 bond^21^. It does not require the formation of the enzyme-plasminogen activator complex, as is the case for streptokinase^21^. The structure of the enzyme is such that upon entrapment it is likely to be oriented with more hydrophobic growth-factor like domain and kringle domains^22^ towards the matrix, which ensures more energetically favorable state, whereas the hydrophilic and positively charged catalytic domain (matrix is also positively charged, see Fig. 1b) faces the pores and, accordingly, solvent molecules, providing the possibility of interaction with the substrate^22^. The size of the enzyme is complementary to the matrix pore size (urokinase size is 5 × 5 × 8 nm, average matrix pore size is 8 nm, see ESI, Fig. 1S for N_2_ physisorbtion data). The plasminogen activation process by the resulting composite could be described as follows: At neutral pH values, negatively charged plasminogen (isoelectric point (IP) = 6.2)^23^ interacts with the catalytic domain of urokinase, located within the positively charged matrix (IP = 8, Fig. 1b), and yields positively charged plasmin (IP = 7.4)^23^, which is repulsed from the matrix, making way for a new substrate molecule. So the composite matrix acting as a conveyor for plasminogen activation and maintain the clot lysis process without any changes in its structure and composition. This mechanism of thrombolytic nanocomposite action is typical for urokinase contents of no more than 12.5 wt%, which corresponds to an enzyme content of 0.5 kU/mg. Below this concentration limit the enzyme is completely entrapped and does not release from the sol-gel matrix, as confirmed by spectral analysis (Fig. 1c) and a lack of enzymatic activity in wash solutions (see experimental details).

The ability of the composite to activate plasminogen can be readily tested by reaction with a chromogenic substrate: activated plasmin can cleave For-Ala-Phe-Lys-pNA yielding colored *p-*nitrophenol (for details see experimental part, experimental data presented at ESI, Fig. 2S)^24^. According to the analysis, the formation of plasmin on a thrombolytic composite (12.5% uPA@ferria) is approximately twice as slow as that for a similar amount of free enzyme, however, this is quite a high index for a heterogeneous system apparently promoted by the correct orientation of urokinase molecule in a thrombolytic composite.

To determine thrombolytic properties of the composite materials, experiments were carried out with an artificial clot derived from human plasma (see experimental details). Using a Mayer rod, a layer of a thrombolytic composite material was coated on a glass slide, resulting in a nanolayer with a thickness of ~200 nm upon drying (Fig. 2a). A clot formed from human plasma was placed on top of the produced thrombolytic coating (basic structure is shown in Fig. 2b) to investigate thrombolysis process (Fig. 2c). While on the surface of a thrombolytic coating, the clot begins to decompose, which can be clearly seen using an optical microscope (Fig. 2d–f). The process of destruction clearly indicates that the thrombolytic coating generates plasmin over its entire surface, which promotes destruction of the clot not only on the perimeter, but also from its center. According to the images, destruction of the clot is characterized by its fragmentation, with an increase in the contact area between the surface of plasmin and fibrin network, which results in faster thrombolysis over time.

Experiments have revealed that the dissolution rate directly depends on the amount of entrapped enzyme (Table 1). Increasing the mass fraction of urokinase in the composite results in an increase in clot destruction rate up to a concentration of 12.5 wt%, which corresponds to an enzyme content of 0.5 kU/mg, while at higher quantities partial release of the thrombolytic enzyme occurred which accelerated the lysis process, but contradicted to the suggested concept. In full accordance with the proposed concept, after washing out residues of the destroyed clot from the thrombolytic coating and placing a new one on top, one also observes destruction of the latter with comparable rate, since thrombolytic activity of the composite material is not associated with the release of plasminogen activator from the matrix, and the resulting material can be used repeatedly or continuously as a thrombolytic coating for cardiovascular implants, e.g., similarly to that used in ref. 18.

Proof of the efficacy of a thrombolytic magnetic composite allowed to move on to producing a colloidal solution of magnetic thrombolytic nanoparticles, which later can be used for parenteral administration. So, the next step implied the creation of thrombolytic nanoparticles acting in the same manner. To this end, the resulting nanocomposite material was mechanically crushed and filtered, so that it contained particles with a size of less than 200 nm, which is the limit permitted for intravenous administration^25^. In this finely dispersed state, thrombolytic enzyme content in nanoparticles can be maintained at a level of up to 10 wt.% (0.4 kU/mg), at larger values the release of the enzyme is observed (for details of experimental results see ESI, Fig. 3S). This phenomenon can be explained by the fact that upon grinding the xerogel matrix the destruction follows the weakest points, i.e., the largest pores, and thus the stripped enzyme is readily released into the aqueous phase. To allow for the parenteral use of the resulting system, particles larger than 200 nm should be filtered using filter nozzles (see experimental part). Next, we consider thrombolytic properties of the produced nanoparticles.

Figure 3a,b show the appearance of nanocomposites before and after grinding and subsequent colloidation to a size of less than 200 nm (Fig. 3c). The resulting hydrosol of thrombolytic nanoparticles with a concentration of 1 wt.% and 4 kU/mL was tested in a number of experiments and manifested itself as an active magnetically controlled thrombolytic agent. Experiments with clots on a slide showed that the nanocomposite has thrombolytic effect only when it is induced by external field of the magnet to an artificial clot, the rate of decomposition being commensurate with that for a thrombolytic coating (120 minutes to completely decompose a clot with a composite with 10 wt% of urokinase, for experimental results see ESI, Fig. 4S), and essentially ineffective if it is located in remote areas due to weak diffusion of plasmin in a liquid layer. For a more detailed study on the mechanism of thrombolysis, we have investigated SEM images of clots treated by magnetic nanoparticles (Fig. 3b–f, see experimental part for details). It is clearly seen that after 15 minutes of clot treatment magnetic nanoparticles begin to form lysed local areas. It is notable how the nanoparticles destroy the clot: the pictures clearly demonstrate how under the influence of nanoparticles the clot begins to decompose in some distance from them, which clearly indicates the reason for the decomposition and is fully consistent with the proposed mechanism. With increasing exposure time the structure becomes more porous, more fibrin strands are destroyed and eventually the clot is dissolved (for process visualization see ESI, Fig. 4S). However, it should be noted that not all nanoparticle aggregates lyse clots, since many areas, in which the nanoparticles are present, do not have a typical lysed shell after a certain period of time. This can occur due to several factors: partial denaturation of the enzyme, incorrect orientation of the catalytic center with respect to the substrate, absence of enzyme molecules, etc.

Thrombolytic nanoparticles also manifested themselves as active for near-real systems. Human blood clots were selected as model systems. To measure the rate of decomposition of thrombi, a static method was used, in which the formed clots were treated by a thrombolytic composite in a suspension with a concentration of 1 kU/mL (Fig. 4a) condensed by a magnet (Fig. 4b). A clot in saline solution (Fig. 4c) and a clot treated with an equivalent amount of urokinase solution (Fig. 4d) were taken as control systems. The process of decomposition was monitored spectrophotometrically by measuring the absorbance at 515 nm. The points were taken in the range from 0 to 90 minutes with a step of 30 minutes. As can be seen from the figures, in the absence of a thrombolytic agent the clot destruction essentially does not proceed, whereas the presence of plasminogen activators promotes the intensive fibrin network destruction and release of erythrocyte residues, which colors the solution. It should be noted that although an uncondensed thrombolytic composite is approximately four times less active than the free enzyme, its condensation on a blood clot significantly accelerates the rate of lysis, increasing it to an average of 250% at the same concentration of the enzyme. For applications in larger volumes, the efficiency of magnetically controlled systems will increase proportionally to a decrease in the concentration of dissolved free enzyme and, when used in the human body with an average blood flow volume of 5 L, can be up to 4000 times higher (see experimental details for calculations) than that for the free enzyme due to the ability to localize the drug at a certain point.

To confirm the efficiency of the system in conditions close to real life, we have carried out experiments on real human blood clots, since these will be the target of thrombolytic therapy. The thrombolytic system was tested on a real human thrombus (Fig. 4e) from an *arteria carotis communis* fragment removed during endarterectomy. The thrombolytic composite concentrated by a magnet promoted the destruction of the clot, which clearly indicates applicability of solid-state thrombolytic composite material in real systems. The actual thrombus decomposition rate was slightly lower than that prepared from blood, which can be explained by the more complex structure of the real system compared to the model one.

This article is the first report on a new class of solid-state thrombolytic systems with external magnetic control characterized by high stability and biocompatibility, which logically follows the previously described development of thrombolytic sol-gel systems. In its mechanism of action the validity of such composites is not limited by the amount of active enzyme in the composite, since the functionality is not associated with the release of the enzyme. Due to the nanoporous architecture and organization of the composite material the catalytic domain of entrapped urokinase can interact with plasminogen, which, on the one hand, leads to the formation of plasmin and, on the other, prevents the release of urokinase from a magnetite matrix. Such materials are able to function for a long time, since the concentration of plasminogen activator does not change over time. When in blood flow, magnetic thrombolytic particles can subsequently be localized at different locations without loss of catalytic activity. This feature is especially important when it comes to floating and decomposing blood clots, for which conventional surgical methods are essentially futile. As evidenced by *in vitro* experiments, the composite was 2.5 times more active than free urokinase due to localization by an external magnetic field. While scaling the system on human blood volume, the efficiency of a magnetically controlled composite can be up to 4000 times higher than that for free enzyme due to the ability to localize the drug at a certain point, thus correspondingly reducing the drug dosage and side effects.

## Experimental Details

### Materials

#### Chemicals

The hydrosol was prepared from iron (II) chloride tetrahydrate, iron (III) chloride hexahydrate and ammonia, all from Sigma-Aldrich. Recombinant urokinase (Purolase©), 100 kU/mL with a molecular weight of 49.3 kDa, was obtained from Russian Cardiology Research and Production Complex. Lyophilized human plasma and human thrombin (150 NIH units/mg), chromogenic substrate for plasmin (For-Ala-Phe-Lys-pNA) and saline solution were obtained from “Kvik” LTD Company.

#### Preparation of a ferria hydrosol

Pure ferria hydrosol was prepared ultrasonically from iron (II) chloride tetrahydrate and iron (III) chloride hexahydrate as described in ref. 19. The mass fraction of the magnetite nanoparticles with an average particle size of 10 nm in sol was 2 wt%.

#### Preparation of thrombolytic coatings

200 μL of freshly prepared hydrosol was treated with 0 to 10 μL of urokinase solution (100 kU/mL) with an enzyme mass fraction of 5 wt% and stirred for 5 minutes. Using a Mayer rod, the resulting mixture was applied on a slide as a 6 micron-thick layer and dried in a vacuum desiccator to yield composite materials with enzyme contents from 0 to 12.5 wt%. The coating obtained was washed with 3 mL of distilled water, and enzyme activity of wash solutions was evaluated as described below. To evaluate the enzyme release, a similar amount of the mixture was dried in a quartz cuvette, treated with 2 mL of distilled water and analyzed by taking a 210 nm absorption spectrum over time upon constant stirring for 12 hours. Enzymatic activity of wash water was evaluated by reaction with a chromogenic substrate, as described in more detail below (*Evaluating enzymatic activity of urokinase*).

#### Preparation of uPA@ferria thrombolytic nanoparticles

A Petri dish containing 200 μL of freshly prepared sol was mixed with 0 to 10 μL of urokinase solution (100 kU/mL). After stirring, the contents were dried in a vacuum desiccator. The resulting composite material was crushed in an agate mortar, suspended in deionized water and passed through a Phenex 200 nm syringe filter to eliminate all particles larger than 200 nm. The resulting suspension was concentrated on a rotary evaporator under reduced pressure at 20 °C and suspended in saline solution with a final solid concentration of 1 wt% of enzyme (0.5 kU/mg solid) and used in experiments. To evaluate the release of the enzyme, the same amount of crushed composite in a quartz cuvette was treated with 2 mL of saline solution and studied by taking a 210 nm absorption spectrum at 37 °C over time. Enzymatic activity of wash water was evaluated by reaction with a chromogenic substrate, as described in more detail below.

#### Preparation of clots from human plasma

Model clots were prepared from control human plasma with known amounts of fibrinogen and plasminogen. 10 μL of standard human plasma solution (plasminogen concentration was 102 μg/mL and that for fibrinogen 2.8 mg/mL) was treated with 10 μL of thrombin solution (150 U/mL). The resulting mixture was gently stirred and allowed to stand for 5 minutes. The formed clot was separated and used in experiments.

#### Obtaining clots from human blood

Model clots were prepared from non-stabilized human blood. 100 μL of human blood (fibrinogen 6.92 g/L and prothrombin Quick index 83.575) was treated with 10 μL of thrombin solution (150 U/mL). The resulting mixture was stirred for 5 minutes by shaking, held for 60 minutes at a temperature of 37 °C, and then kept for 5 hours at 4 °C for the final formation. The formed artificial blood clot was separated, washed with saline solution and used in experiments.

#### Human thrombus

Human thrombus were obtained from a 66-year-old man as a result of *arteria carotis interna* thromboendarterectomy surgery due to critical stenosis up to 90% of the lumen and acute thrombosis (for visual appearance see ESI Fig. 5S). The operation was carried out in a single step under endotracheal anesthesia on a patient (U., 66) who was admitted to a vascular center with acute ischemic stroke. The post-operative thrombus was untreated and used for the next 48 hours.

## Methods

All experiments on blood and tissue samples were approved by institutional ethical committee (Mariinskiy Hospital, №0915) and are in agreement with the guidelines approved by the Government of Russian Federation Ministry of Health N 346N of 12 may 2010. These guidelines follow the *Directive 2004/23/EU* and *2002/98/EU* of the European Parliament on usage of human tissues and blood samples for scientific purposes. Informed signed consent was obtained from the patients to perform experiments on related biomaterials. Written informed consent was obtained for publication of identifying information relating to participants.

### Characterization methods

Specific surface area, pore volume and pore size distribution were investigated using Quantachrome Nova 1200e by nitrogen adsorption at 77 K and analyzed by the BET and BJH equations. Prior to analysis, all samples were degassed at room temperature for 48 hours. The samples for transmission electron microscopy (TEM) were obtained by dispersing a small probe in ethanol to form a homogeneous suspension. Then, a suspension drop was coated on a copper mesh covered with carbon for a TEM analysis (FEI TECNAI G2 F20, at an operating voltage of 200 kV). To analyze the samples using high-resolution scanning electron microscopy (SEM), the obtained ground xerogel was deposited on a metal tip and investigated without additional spraying using a Magellan 400 L ultra-high resolution electron microscope. To analyze plasma clot using SEM, the obtained samples were dried under vacuum for 1 hour and investigated without additional spraying using a TESCAN VEGA 3 electron microscope. Optical microscopy was done on a LOMO MIKMED 6 microscope with an X10 lens. Hydrodynamic diameter was measured by the DLS technique on Photocor Compact Z. Spectrophotometrical measurements of enzymatic activity were carried out using an Agilent Cary HP 8454 Diode Array spectrophotometer with TEC.

### Evaluating enzymatic activity of uPA

Activity of the free uPA and uPA@ferria composite was evaluated by measuring the level of generated plasmin. 1 mL of the sample was incubated with 100 μL of standard human plasma with a known amount of plasminogen (102 μg/mL) for 10 minutes at 37 °C, followed by addition of 0.5 mL of a chromogenic substrate with a concentration of 3 mg/mL and incubated for 120 seconds at 37 °C, after which 1 mL of 30% acetic acid was added and absorbance was measured at 405 nm. In tests on the thrombolytic composite ability to activate plasminogen, the time for incubating the composite with plasma varied from 5 to 25 minutes.

### Thrombolytic activity of uPA@ferria films

The activity of uPA@ferria films was studied using an optical microscope The clots were formed as described above with a subsequent deposition on the thrombolytic sol-gel coatings formed on a glass slide. Transmission mode was used to see any morphological changes in clots during analysis. The thrombolysis was monitored as a function of time. The thickness of the clots was controlled with a 10 μm copper foil clamped between glasses. The pictures were taken every 5 minutes with a subsequent analysis.

### Thrombolytic activity of uPA@ferria nanoparticles

Thrombolytic activity of the composite was studied using an optical microscope. A plasma clot formed by the technique described above was placed on a glass slide, with 100 μL of a thrombolytic system sol (with an activity of 4 kU/mL) obtained by the method described above added to it, and then treated by concentrating the magnetic particles in the clot area or at a distance of 20 mm from it. Transmission mode was used to see any morphological changes in clots during analysis. The thrombolysis was monitored as a function of time. The thickness of clots was controlled with a 10 μm copper foil clamped between glasses.

### Investigating thrombolytic activity of uPA@ferria nanoparticles using SEM

A plasma clot formed using the technique described above was applied to a metal substrate, with 100 μL of a thrombolytic system sol (with an activity of 4 kU/mL) obtained by the method described above added to it, and incubated at 37 °C for 15 minutes. The treated clot was dried in a vacuum desiccator for 1 hour, after which its morphology was studied using an electron microscope.

### Blood clot lysis

A blood clot formed using the method described above was placed in a cuvette with 3 mL of saline solution added to it. The cuvette was then treated with 100 μL of thrombolytic composite solution (activity 4 kU/mL) and studied by taking a 515 nm absorption spectrum at a temperature of 37 °C in kinetic mode. To perform a control experiment, the formed clot was placed in a cuvette containing 3 mL of saline solution and studied over time by taking a 515 nm absorption spectrum at a temperature of 37 °C every 30 minutes for 90 minutes.

### Human blood clot lysis

A 5 cm^2^ excised fragment of *arteria carotis interna* was placed in a cuvette, with 3 mL of saline and 100 μL of thrombolytic composite (activity 4 kU/mL) solutions added to it, and studied by taking a 515 nm absorption spectrum at a temperature of 37 °C every 30 minutes for 90 minutes.

### Supporting calculations

Experiments were carried out in a volume of 3 mL. If the volume increases to 5 L (mean adult blood volume), urokinase concentration will proportionally drop 1600-fold. A magnetic thrombolytic composite could be magnetically concentrated in a desired spot by external magnetic field, so the local concentration of the composite will not be affected. In a 3 mL cuvette, thrombolytic activity of a concentrated composite is 250% higher than of the enzyme solution, so activity of a magnetically concentrated composite would be 1600 × 2.5 = 4000 times higher.

## Additional Information

**How to cite this article**: Drozdov, A. S. *et al*. Leach-proof magnetic thrombolytic nanoparticles and coatings of enhanced activity. *Sci. Rep.* **6**, 28119; doi: 10.1038/srep28119 (2016).

## Supplementary Material

Supplementary Information

## Figures and Tables

**Figure 1:**
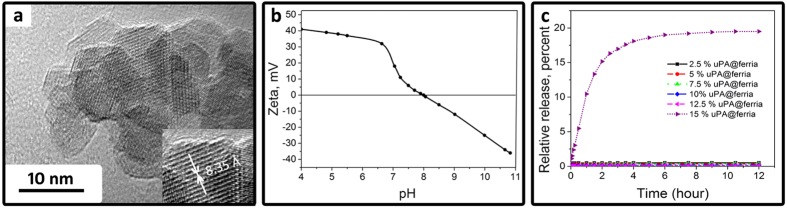
TEM image of the magnetite matrix (**a**), zeta potential of the magnetite nanoparticles as a function of pH (**b**), release of the uPA from the uPA@ferria composite at the different mass fractions.

**Figure 2:**
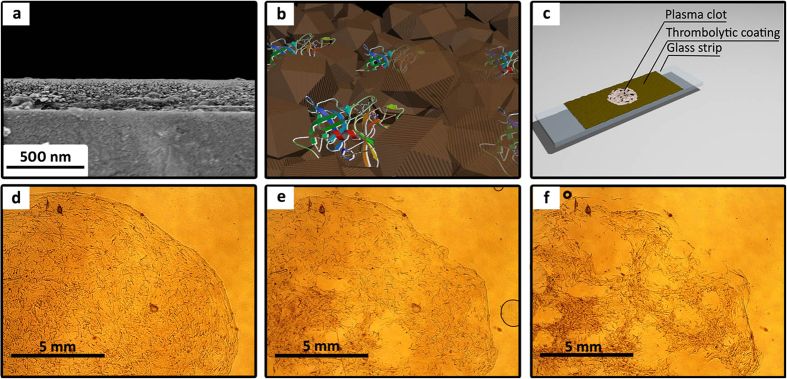
SEM cross-section image of a thrombolytic coating with 12.5 wt% entrapped uPA (**a**); schematic representation of a magnetic thrombolytic composite (MTC) (**b**); scheme for thrombolytic analysis of uPA@ferria films carried out using an optical microscope (**c**); visualization of the plasma clot lysis process provided by MTC using an optical microscope at 0, 45, and 90 minutes, respectively (**d–f**), lens X10.

**Figure 3:**
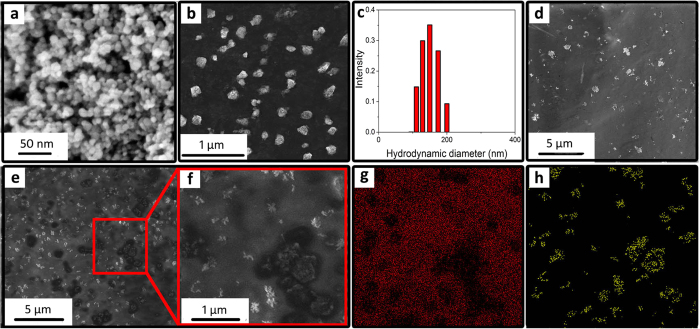
HR SEM image of the nanocomposite (12.5% uPA@ferria) (**a**) and appearance of it after (**b**) grinding and subsequent colloidation; (**c**) thrombolytic nanoparticle size distribution after filtration; clot surface coated with thrombolytic nanoparticles immediately after preparation (**d**); after 15 minutes of treatment with thrombolytic nanoparticles (**e,f**) a magnified area of the clot partially decomposed by thrombolytic nanoparticles with respective EDX image mapping on the carbon (**g**) and iron (**h**) atoms.

**Figure 4:**
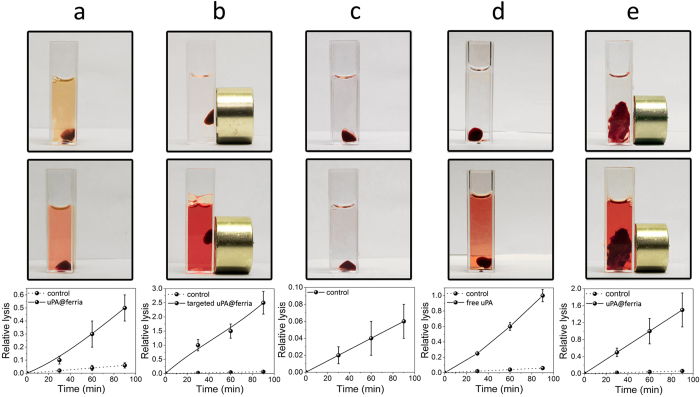
Visual appearance (upper and middle lines) and relative lysis kinetics (bottom line) for a blood clot in the presence of a thrombolytic composite (**a**), magnetically targeted thrombolytic composite (**b**), saline (**c**), and in the presence of a uPA solution (**d**). Lysis of human thrombus in the presence of a magnetically targeted thrombolytic composite (**e**).

**Table 1:** Clot lysis rate vs. enzyme concentration in the matrix.

Material	Full lysis time (first clot), min (±15)	Full lysis time (second clot), min (±15)
ferria matrix	–	–
2.5% uPA@ferria	275	289
5% uPA@ferria	210	224
7.5% uPA@ferria	150	165
10% uPA@ferria	120	110
12.5% uPA@ferria	90	100

## References

[b1] Arboix A. & Alió J. Cardioembolic stroke: clinical features, specific cardiac disorders and prognosis. Curr. Cardiol. Rev. 6, 150–161 (2010). 21804774 10.2174/157340310791658730 PMC2994107

[b2] Marti C. . Systemic thrombolytic therapy for acute pulmonary embolism: a systematic review and meta-analysis. Eur. Heart J. 36, 605–614 (2015). 24917641 10.1093/eurheartj/ehu218 PMC4352209

[b3] Ishihara M. . Long‐term prognosis of late spontaneous reperfusion after failed thrombolysis for acute myocardial infarction. Clin. Cardiol. 22, 787–790 (1999). 10626080 10.1002/clc.4960221206 PMC6655750

[b4] Torno D. . Improvement of *in vitro* thrombolysis employ in magnetically-guided microspheres. Thromb. Res. 121, 799–811 (2008). 17942144 10.1016/j.thromres.2007.08.017

[b5] Westein E., Flierl U., Hagemeyer C. E. & Peter K. Destination known: targeted drug delivery in atherosclerosis and thrombosis. Drug Dev. Res. 74, 460–471 (2013).

[b6] El-Sherbiny M., Elkholi E. & Yacoub H. Tissue plasminogen activator-based clot busting: Controlled delivery approaches. Glob. Cardiol. Sci. Pract. 3, 336–349 (2014). 25780787 10.5339/gcsp.2014.46 PMC4352685

[b7] Kaminski D. . Encapsulation and release of plasminogen activator from biodegradable magnetic microcarriers. Eur. J. Pharm. Sci. 35, 96–103 (2008). 18644439 10.1016/j.ejps.2008.06.012

[b8] Ma H. . Magnetically targeted thrombolysis with recombinant tissue plasminogen activator bound to polyacrylic acid-coated nanoparticles. Biomaterials 30, 3343–3351 (2009). 19299010 10.1016/j.biomaterials.2009.02.034

[b9] Voros E. . TPA immobilization on iron oxide nanocubes and localized magnetic hyperthermia accelerate blood clot lysis. Adv. Funct. Mat. 25, 1709–1718 (2015).

[b10] Zhou J. . Construction and evaluation of Fe_3_O_4_-based PLGA nanoparticles carrying rtPA used in the detection of thrombosis and in targeted thrombolysis. ACS Appl. Mater. Interfaces 6, 5566–5576 (2014). 24693875 10.1021/am406008k

[b11] Cheng R. . Acceleration of Tissue Plasminogen Activator-Mediated Thrombolysis by Magnetically Powered Nanomotors. ACS Nano 8, 7746–7754 (2014). 25006696 10.1021/nn5029955 PMC4148143

[b12] Davoodi . Coaxial electrohydrodynamic atomization: Microparticles for drug delivery applications. J. Controlled Release 205, 70–82 (2015). 10.1016/j.jconrel.2014.12.004 25483422

[b13] Drozdov A., Volodina K., Vinogradov V. & Vinogradov V. V. Biocomposites for wound-healing based on sol–gel magnetite. RSC Adv. 101, 82992–82997 (2015).

[b14] Yang W. . Bioconjugation of Recombinant Tissue Plasminogen Activator to Magnetic Nanocarriers for Targeted Thrombolysis. Int. J. Nanomed. 7, 5159–5173 (2012). 10.2147/IJN.S32939 PMC3464084 23055728

[b15] Chen P., Yang C., Ma H. & Wu T. Characterization of Chitosan Magnetic Nanoparticles for *in Situ* Delivery of Tissue Plasminogen Activator. Carbohydr. Polym. 84, 364–372 (2011).

[b16] Chen P., Yang C., Ma H., Tu J. & Lu J. Targeted Delivery of Tissue Plasminogen Activator by Binding to Silica-Coated Magnetic Nanoparticle. Int. J. Nanomed. 7, 5137–5149 (2012). 10.2147/IJN.S36197 PMC3463402 23055726

[b17] Vinogradov V. V., Vinogradov A. V., Sobolev V. E., Dudanov I. P. & Vinogradov V. V. Plasminogen activator entrapped within injectable alumina: a novel approach to thrombolysis treatment. J. Sol-Gel Sci. Technol. 73, 501–505 (2015).

[b18] Chapurina Y. . Synthesis of Thrombolytic Sol-Gel Coatings: Toward Drug-Entrapped Vascular Grafts. J. Med. Chem. 58, 6313–6317 (2015). 26199987 10.1021/acs.jmedchem.5b00654

[b19] Drozdov A., Ivanovski V., Avnir D. & Vinogradov V. A universal magnetic ferrofluid: Nanomagnetite stable hydrosol with no added dispersants and at neutral pH. J. Colloid Interface Sci. 468, 307–312 (2016). 26852355 10.1016/j.jcis.2016.01.061

[b20] Drozdov A., Shapovalova O., Ivanovski V., Avnir D. & Vinogradov V. V. Entrapment of enzymes within sol–gel-derived magnetite. Chem. Mater. 28, 2248–2253 (2016).

[b21] Cesarman‐Maus G. & Hajjar A. Molecular mechanisms of fibrinolysis. Br. J. Haematol. 129, 307–321 (2005). 15842654 10.1111/j.1365-2141.2005.05444.x

[b22] Huai Q. . Structure of human urokinase plasminogen activator in complex with its receptor. Science 311, 656–659 (2006). 16456079 10.1126/science.1121143

[b23] Robbins K. C. & Summaria L. Plasminogen and plasmin. Methods in Enzymol. 45, 257–272 (1976). 138064 10.1016/s0076-6879(76)45025-5

[b24] Stepanov V. M., Terentyeva E. Yu., Voyushina T. L. & Gololobov M. Yu. Subtilisin and α-chymotrypsin catalyzed synthesis of peptides containing arginine and lysine p-nitroanilides as c-terminal moieties. Bioorg. Med. Chem. 3, 479–485 (1995). 7648197 10.1016/0968-0896(95)00073-p

[b25] Champion J. A., Katare Y. K. & Mitragotri S. Particle shape: a new design parameter for micro- and nanoscale drug delivery carriers. J. Controlled Release 121, 3–9 (2007). 10.1016/j.jconrel.2007.03.022 PMC4009069 17544538

