# Supplementary material for: Leach-proof magnetic thrombolytic nanoparticles and coatings of enhanced activity
Source: Sci Rep. 2016 Jun 20;6:28119. doi: 10.1038/srep28119 (PMC4913305; doi:10.1038/srep28119)
Supplement: Supplementary Information [file srep28119-s1.pdf]

Supplementary data for

Leach-proof magnetic thrombolytic nanoparticles and coatings of enhanced activity

By Andrey S. Drozdov[1], Vasiliy V. Vinogradov[1], Ivan P. Dudanov[1,2], and Vladimir V. Vinogradov[1]*

[1]ITMO University, Laboratory of Solution Chemistry of Advanced Materials and Technologies,
Lomonosova St. 9, 191002, St. Petersburg, Russian Federation
E-mail: vinogradov@scamt.ru

[2]Mariinskiy Hospital, Regional Cardiovascular Center, Liteyny Ave. 56, 191054, St. Petersburg, Russian Federation

Isotherm of nitrogen adsorption and average pore diameter

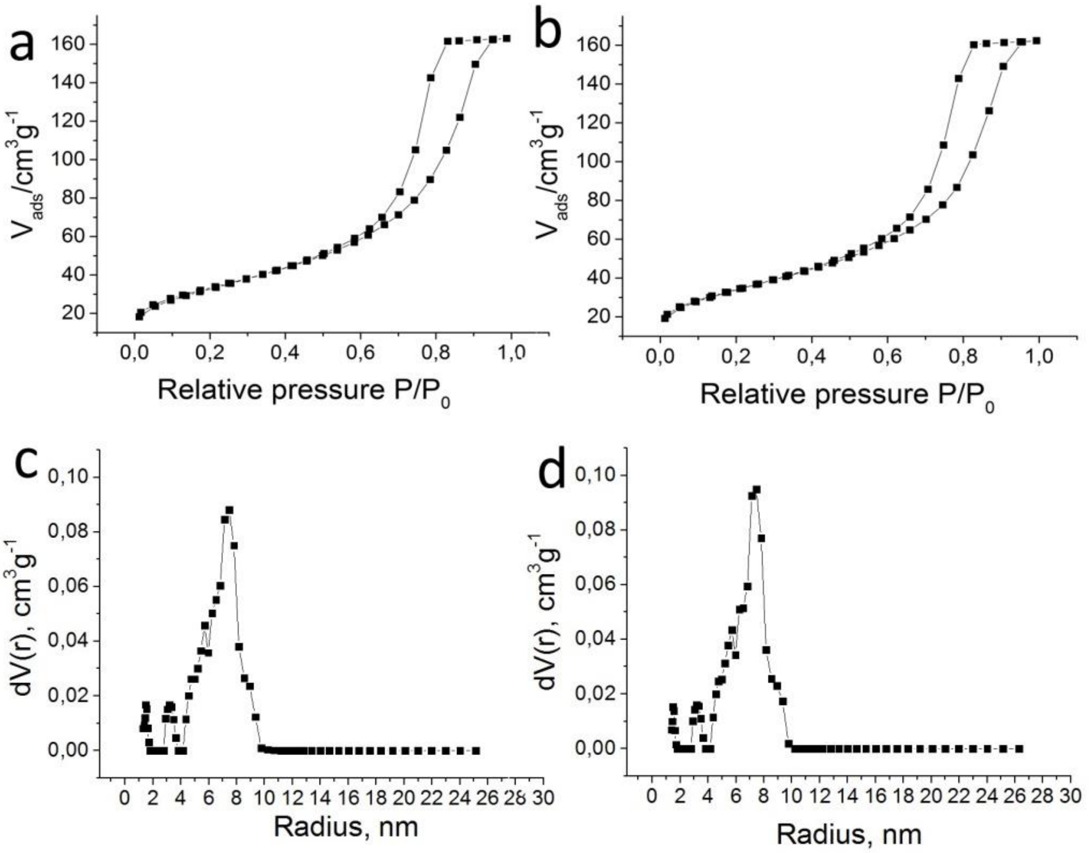

Figure 1S. Nitrogen adsorption-desorption isotherm for a magnetite matrix (a); nitrogen adsorption-desorption isotherm for a thrombolytic composite with 10 wt% of urokinase (b); pore size distribution for a magnetite matrix (c); pore size distribution for a thrombolytic composite with 10 wt% of urokinase (d).

Analysis of nitrogen adsorption-desorption isotherms using the BJH method shows that the pore sizes for a free matrix and a matrix with entrapped enzyme are essentially the same. Pore size (8 nm) is perfect for entrapping urokinase enzyme with a size of 5 × 5 × 8 nm.

Formation of plasmin by a thrombolytic material

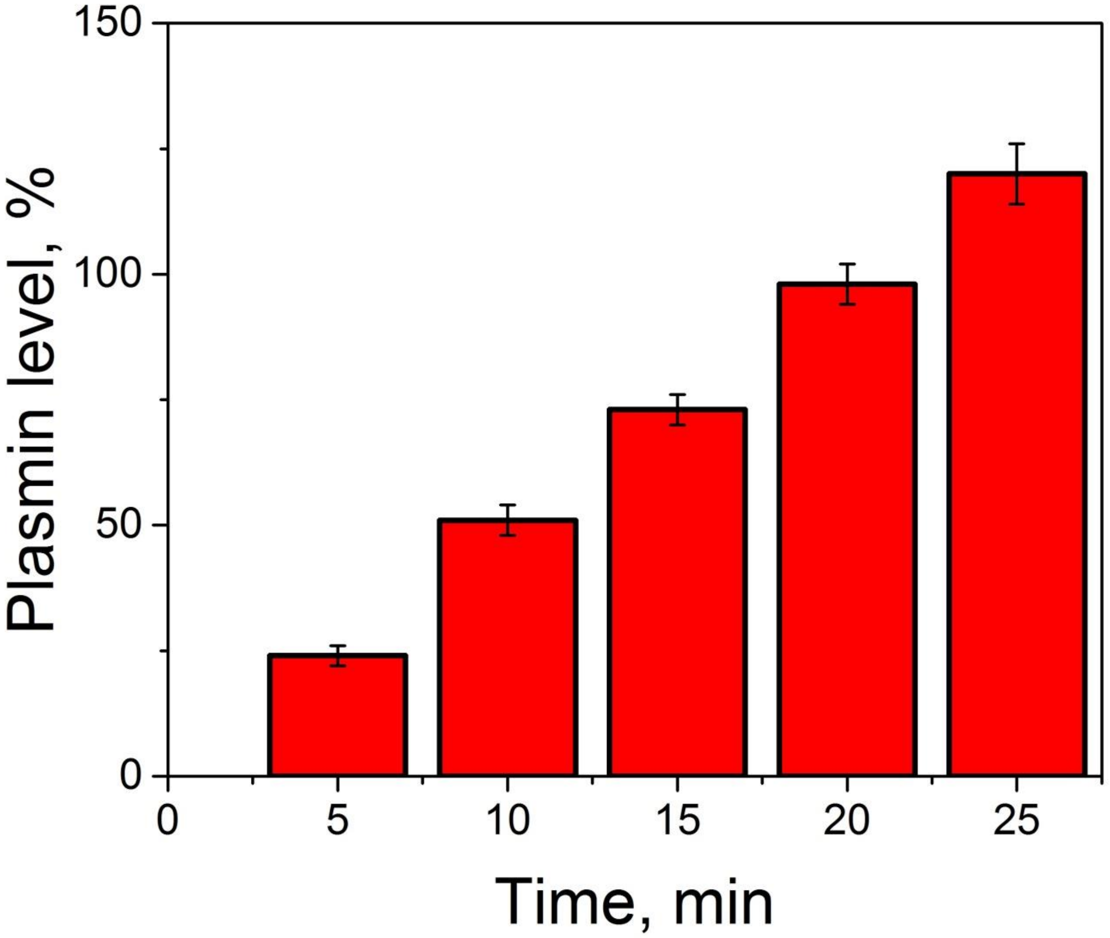

Figure 2S. Level of plasmin formed by a thrombolytic composite with 12.5 wt% of urokinase as measured by a reaction with a chromogenic substrate.

The composite was incubated with control plasma with a known level of plasminogen for different periods of time, after which the level of formed plasmin was measured by its reaction with a chromogenic substrate. The level of plasmin formed by the same amount of free urokinase for 10 minutes is taken as 100%.

Profile of urokinase release from a magnetite nanocomposite

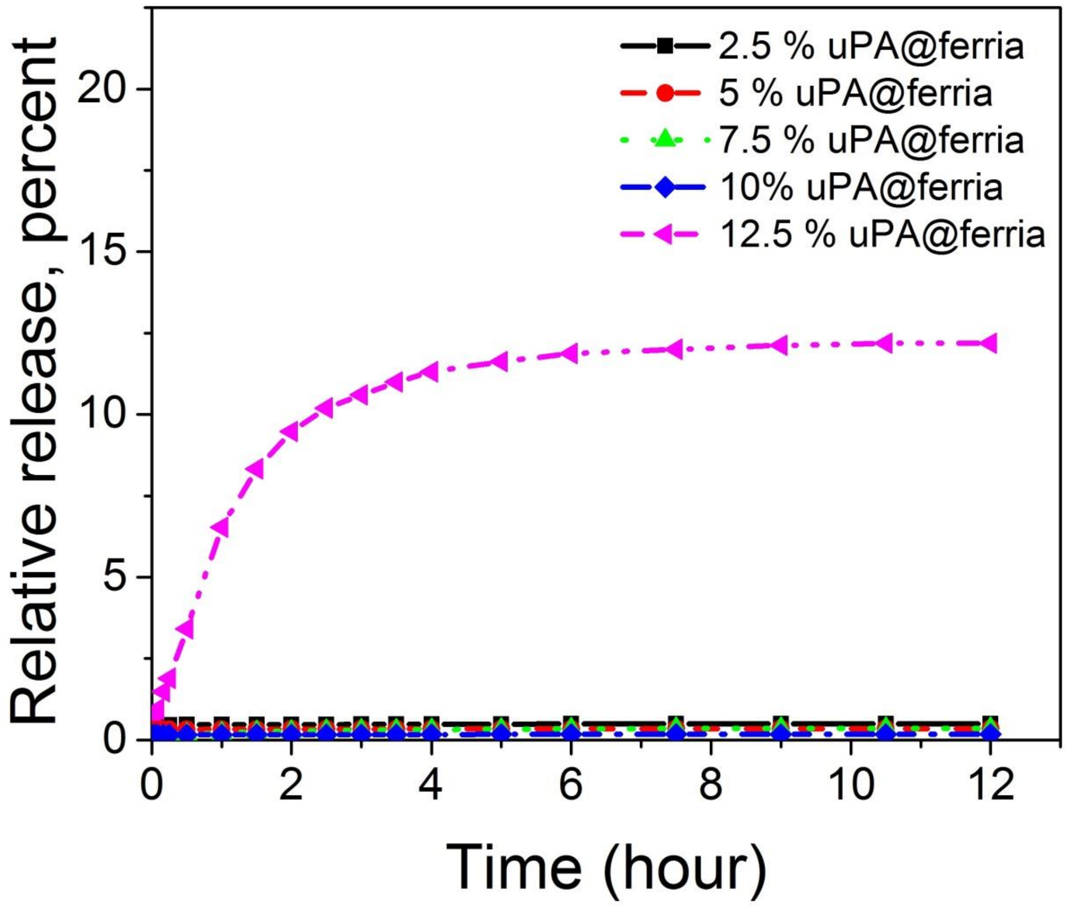

Figure 3S. Profile of urokinase release from a dispersed nanocomposite hydrosol.

For enzyme concentrations lower than 10 wt%, the absence of release is seen. At a concentration of 12.5 wt% urokinase starts to release from the matrix, which renders such composites inappropriate for the used conception.

Visualization of the plasma clot lysis process provided by thrombolytic nanoparticles using an optical microscope

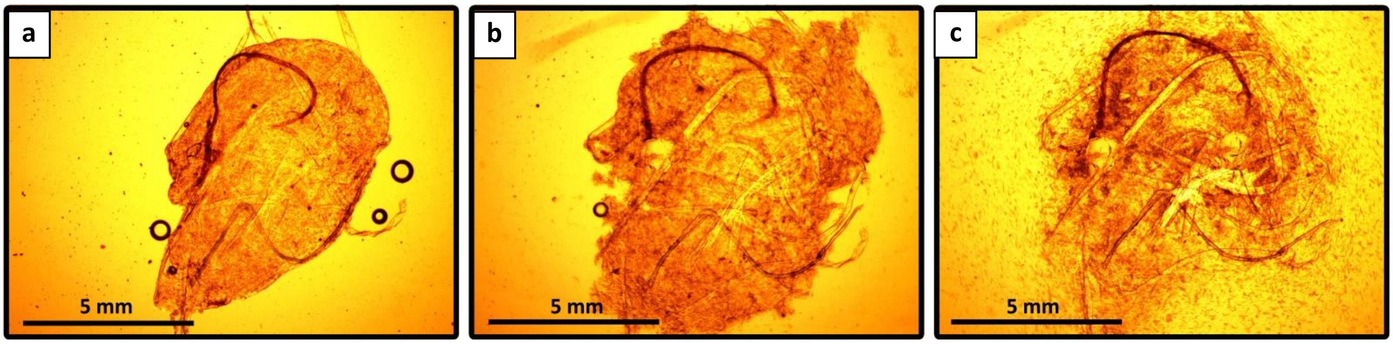

Figure 4S. Plasma clot lysis with magnetically targeted nanocomposite.

Visualization of the plasma clot lysis process provided by magnetically targeted thrombolytic nanoparticles with a 10% urokinase loading using an optical microscope. Pictures are taken at 0 (a), 60 (b) and 120 (c) minutes after targeting.

<u>Extraction of human biosamples</u>

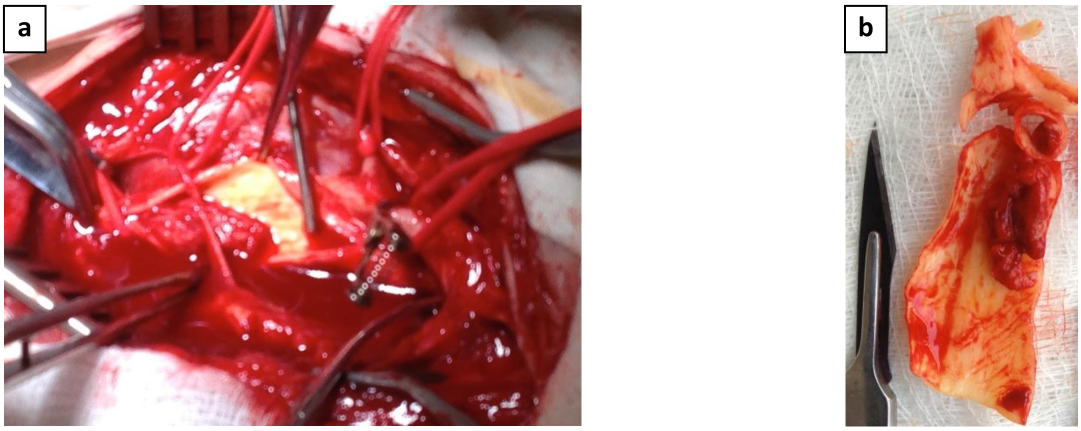

Figure 5S. Open *arteria carotis interna* thromboendarterectomy surgery on a 66-year-old patient. Operations arteriotomy, thrombointimectomy, removal of an occlusive thrombus and an atherosclerotic plaque of the carotid artery (a) an excised plaque sample, with intimal dissection (at the upper pole of the clot) and parietal thrombus (b).
